# The Nephrotoxin Puromycin Aminonucleoside Induces Injury in Kidney Organoids Differentiated from Induced Pluripotent Stem Cells

**DOI:** 10.3390/cells11040635

**Published:** 2022-02-11

**Authors:** Lisa Nguyen, Wasco Wruck, Lars Erichsen, Nina Graffmann, James Adjaye

**Affiliations:** Institute of Stem Cell Research and Regenerative Medicine, Medical Faculty, Heinrich-Heine University, 40225 Dusseldorf, Germany; lisa.nguyen@med.uni-duesseldorf.de (L.N.); wasco.wruck@med.uni-duesseldorf.de (W.W.); lars.erichsen@med.uni-duesseldorf.de (L.E.); nina.graffmann@med.uni-duesseldorf.de (N.G.)

**Keywords:** urine cells, iPSCs, organoids, puromycin aminonucleoside, AKI, inflammation, apoptosis, DNA damage, RAAS

## Abstract

Kidney diseases, including acute kidney injury (AKI) and chronic kidney disease (CKD), which can progress to end stage renal disease (ESRD), are a worldwide health burden. Organ transplantation or kidney dialysis are the only effective available therapeutic tools. Therefore, in vitro models of kidney diseases and the development of prospective therapeutic options are urgently needed. Within the kidney, the glomeruli are involved in blood filtration and waste excretion and are easily affected by changing cellular conditions. Puromycin aminonucleoside (PAN) is a nephrotoxin, which can be employed to induce acute glomerular damage and to model glomerular disease. For this reason, we generated kidney organoids from three iPSC lines and treated these with PAN in order to induce kidney injury. Morphological observations revealed the disruption of glomerular and tubular structures within the kidney organoids upon PAN treatment, which were confirmed by transcriptome analyses. Subsequent analyses revealed an upregulation of immune response as well as inflammatory and cell-death-related processes. We conclude that the treatment of iPSC-derived kidney organoids with PAN induces kidney injury mediated by an intertwined network of inflammation, cytoskeletal re-arrangement, DNA damage, apoptosis and cell death. Furthermore, urine-stem-cell-derived kidney organoids can be used to model kidney-associated diseases and drug discovery.

## 1. Introduction

Kidney diseases such as acute kidney injury (AKI) and chronic kidney disease (CKD) are a worldwide health problem. While AKI describes a sudden loss of renal function and can be caused by diverse conditions such as sepsis, nephrotoxicity or ischemia-reperfusion injury (IRI) [1], CKD is defined as a gradual deprivation of kidney function over a time period of more than three months [2]. Major causes of CKD include diabetes and hypertension as well as oxidative stress and inflammation [2,3]. The progression of CKD leads to end-stage renal disease (ESRD) requiring dialysis or organ transplantation [4]. Besides CKD and AKI, various conditions such as diabetes, hypertension and obesity can lead to the emergence of ESRD [5].

Hypertension may result in damage of the glomeruli in the kidney, as an increased hydraulic pressure disrupts the delicate glomerular capillary system [6]. The glomerulus is an organ compartment, essential for blood filtration and the excretion of toxins. The glomerular barrier consists of a basement membrane, endothelial cells and intertwined podocytic foot processes [5]. Part of the filtration barrier is formed by slit diaphragms, assembled by podocyte-associated proteins nephrin, podocin, synaptopodin, CD-2-associated protein (CD2AP) and the zonula occludens protein-1 (ZO-1) [7]. In many cases, the main target of various kidney diseases, including minimal change disease (MCD) and focal segmental glomerulosclerosis (FSGS), are glomerular podocytes [8]. Moreover, various nephrotoxic substances lead to podocyte injury [5,7].

One such nephrotoxic substance is puromycin aminonucleoside (PAN), a purine antagonist, which is classified as an antibiotic, antineoplastic and antimetabolite substance [7]. It inhibits RNA synthesis and is known to induce acute glomerular damage. Interestingly, other than rats, monkeys and humans, no other species is affected by PAN nephrotoxicity [7]. It was first shown in 1990 that PAN affects and alters the ultrastructure of rat glomerular podocytes of kidney slices, leading to a decrease in or loss of microvilli, flattening of the podocyte cell bodies and the emergence of membranous blebbing [9]. 

To date, human models of kidney diseases are sparse. As most primary cells lose their functionality and viability in vitro, numerous kidney diseases were modelled in rodents [10,11]. However, as murine models do not accurately recapitulate the human disease conditions, the knowledge gained from this research cannot be completely extrapolated to human kidney-associated diseases [10].

Besides improving rodent models, the establishment of pluripotent stem-cell-based kidney cells in 2D or 3D organoids holds great potential for gaining further insights into disease mechanisms and potential therapies [12]. Recent studies have suggested using primary kidney cells for the generation of kidney organoids as an alternative to pluripotent stem cells [13,14,15]. Kidney organoids are composed of a variety of kidney cell types and fulfil organ functions to a certain degree, which make them the best option for modelling kidney diseases. Approaches to model podocyte injury and possible therapies were made by harnessing PAN treatment on iPSC-derived podocytes and kidney organoids [5].

In this study, we applied puromycin aminonucleoside for the induction of renal damage in our kidney organoids to model kidney injury, and we then used transcriptome-based analyses to identify affected pathways and gene ontologies such as DNA damage and inflammation. 

## 2. Materials and Methods

### 2.1. Cell Cultivation and Formation of Kidney Organoids

Integration-free iPSC lines used for the generation of kidney organoids were urine-cell-derived—UM51 and UF21 [16,17]—and foreskin-fibroblast-derived—B4 [18]. The cells were reprogrammed with episomal plasmids.

Cells were maintained in mTeSR1 medium (Stem Cell) with daily medium change. Single-cell splitting was carried out at 70–80% confluence. The iPSCs were incubated in 0.5% EDTA/PBS for 5–10 min, and the total cell number was determined by counting in a Neubauer Counting Chamber. Approximately 0.2–0.5 × 10^5^ cells per well were seeded into a low-attachment, 96-well plate (Thermo Fisher, Darmstadt, Germany). The plate was centrifuged at 300× *g* for 3 min. After 3–5 days, iPSCs clustered to small, round and dense cell aggregates, which were transferred to non-adherent, bacterial 92 × 16 mm Petri-dishes and placed in a shaking incubator at 37 °C, 5% CO_2_, normoxia and 60 rpm. Undifferentiated iPSC spheroids were maintained in basal spheroid medium (BSM) (see Table S1).

The protocols for differentiating iPSC spheroids towards kidney were adapted from Low et al. [19] with slight modifications. The induction of primitive streak was started by culturing the cells for 4 days in basal differentiation medium (BDM) (see Table S2) supplemented with 10 µM CHIR99021 (Tebu-bio, Offenbach, Germany), which is a GSK3 inhibitor and WNT pathway activator. The medium was changed to BDM without additional supplements to achieve the second stage of intermediate mesoderm for 3 days. The emergence of nephron progenitor cells was induced by incubating the cells with BDM supplemented with 3 µM CHIR99021 and 50 ng/mL FGF9 (peprotech, Cranbury, NJ, USA) for 2 days. Over the course of 5 days, the spheroids were fed with BDM plus 50 ng/mL FGF9 to induce nephrogenesis. Starting from D14, a supplementation with 1 µM of CHIR99021 was applied. Patterning was carried out for 6 days with BDM supplemented with 1 µM of CHIR99021. Kidney organoids were then maintained in unsupplemented BDM until further use. Four independent organoid batches in biological duplicates (see Table 1) were generated. The names of the kidney organoid batches are composed of their origin, the donors’ gender and the replicate number (see Table 1). The FFK1/2 organoids were derived from foetal foreskin and were only named after their cellular origin and the replicate number without the donors’ gender (see Table 1). The organoids were used for kidney injury modelling by induction with the cytotoxic substance puromycin aminonucleoside (PAN) (Sigma-Aldrich, Taufkirchen, Germany). In previous works, we determined the ideal PAN concentration for our kidney organoids (not shown). We tested the concentrations 10, 50 and 100 µg/mL PAN and selected the concentration of 50 µg/mL for subsequent experiments. To induce kidney damages, kidney spheroids were treated with 50 µg/mL PAN for 48 h.

### 2.2. Cryosectioning

Kidney organoids were fixed in 4% Formaldehyde (Polysciences, Warrington, FL, USA) prior to embedding. Dehydration was achieved by washing the cells with distinct concentrations of sucrose solutions. Thereafter, the organoids were placed in moulds filled with TissueTek O.C.T Compound (Sakura Finetek, Umkirch, Germany). 2-methylbutan (Carl Roth, Karlsruhe, Germany) and dry ice were used to snap-freeze the organoids. Sections of 10 µm thickness were prepared with a Cryostat (CM1850, Leica, Nussloch, Germany).

### 2.3. Western Blotting

Total protein from kidney organoids, treated with 50 µg/mL PAN and the specific untreated control, and the undifferentiated iPSC spheroids was extracted with RIPA buffer (Sigma-Aldrich) containing protease and phosphatase inhibitors (Roche, Mannheim, Germany). Protein concentrations were determined using the Pierce BCA Protein Assay Kit (Thermo Fisher). Approximately 20 µg of the protein lysates were separated in a 4–12% SDS-PAGE and the proteins transferred to a nitrocellulose membrane by wet blotting. Membranes were then stained with anti-P53, anti-cleaved Caspase 3 and anti-γH2A.X, and compatible secondary antibodies were used subsequently (for dilutions, see Table S3). Beta-actin was used to normalize protein expression. Protein bands were visualized with ECL Western Blotting Detection Reagents (Cytiva, Freiburg, Germany) and detected in a UV chamber. Band intensity was quantified in the software *Fiji: Image J* (version 1.52a, National Institues of Health, Bethesda, MD, USA) and was normalized to beta-actin levels.

### 2.4. Immunocytochemistry

Frozen sections were thawed to room temperature. TissueTek was washed off with PBS. Organoid sections were encircled with a hydrophobic pen and were incubated in 0.5% Triton/PBS to permeabilize the cells within the sections. Unspecific binding sites were blocked with 3% BSA for 1 h. The primary antibody solution was incubated overnight at 4 °C (see Table S3). After thoroughly washing off the primary antibodies, 1 h incubation with secondary antibodies was conducted. Nuclei were stained with Hoechst. Stained sections were analysed using a Zeiss fluorescence microscope (LSM 700). Particular staining regions were observed under a Zeiss confocal microscope (LSM 700).

### 2.5. Immunohistochemistry and Histology

Proximal tubules within the kidney organoid sections were detected by immunohistochemistry via glycoprotein Lotustetragonolobus lectin (LTL) (Vectorlabs, Burlingame, CA, USA). The histochemistry procedure was conducted following the manufacturer’s instruction manual. Streptavidin Alexa488 antibody (R&D Systems, Bleiswijk, Netherlands) was used to detect LTL. 

Histological images of kidney organoids were prepared with Haematoxylin and Eosin staining. A standard protocol was followed for the histological staining. Tissue structures were imaged under a light microscope. 

### 2.6. Fluorometric Renin Assay

The conditioned media of kidney organoids 48 h after PAN treatment and the specific untreated control were used for the detection of Renin concentration employing the Renin assay kit (Abcam). Samples were prepared in technical duplicates. The fluorometric assay was conducted following the manufacturer’s instructions manual. Fluorescence intensity was measured using the micro plate reader infinite M1000 Pro (Tecan, Grödig/Salzburg, Austria) at Ex/Em = 540/590 nm. The renin standards were plotted in a standard curve and were subsequently used for the determination of renin concentration in the conditioned media of kidney organoids.

### 2.7. In Vitro Dextran Uptake Assay

A 100 µg/mL concentration of Alexa Fluor™ 647-coupled dextran (10.000 MW; Thermo Fisher) was applied to kidney organoids and iPSC spheroids for 4 h. Thereafter, the medium was replenished, and live organoids were visualized under a fluorescence microscope. Kidney organoids and iPSC spheroids were then cultivated for 24 h without dextran and were visualized under a fluorescence microscope. 

### 2.8. qRT-PCR

RNA was isolated from 3-4 kidney spheroids treated with and without PAN as well as the undifferentiated iPSC spheroids using the QIAgen RNeasy Micro Kit. RNA extraction was conducted following the manufacturer’s protocol. In brief, kidney spheroids were lysed in 350 µL of RLT buffer. Supernatant was mixed with an equal volume of 70% ethanol and centrifuged through a gDNA eliminator column. After several washing steps, the RNA was solved in 25 µL of RNAse-free water. A total of 500 ng RNA was used as an input for cDNA synthesis, and qRT-PCR based on SYBR Green was conducted. The primer sequences are presented in Table S4.

### 2.9. Analysis of Gene Expression Data

Duplicates of spheroids and kidney organoids treated with PAN as well as untreated samples were hybridized at the BMFZ (Biomedizinisches Forschungszentrum) core facility of the Heinrich-Heine University (Düsseldorf, Germany) on the Affymetrix Human Clariom S assay. Raw data (CEL files) delivered from the core facility were read into the R/Bioconductor environment for follow-up-processing. The Bioconductor package oligo [20] was employed to correct data for background signals and to normalize data via the Robust Multi-array Average (RMA) method. Via the VennDiagram package [21], Venn diagrams were generated for the dissection of genes annotated uniquely to microarray probesets. Probesets were considered expressed when their detection-*p*-value was below a threshold of 0.05. The detection-*p*-value was calculated by a statistic over designated background probesets on the microarray, as described in Graffmann et al. [22]. With the R hierarchical clustering function *hclust*, a clustering dendrogram of genes with a coefficient of variation greater than 0.1 was produced using Pearson correlation as the similarity measure and complete linkage agglomeration. Heatmaps and associated clustering analyses were generated with the function *heatmap.2* from the R gplots package [23]. Genes that were expressed in at least one of the experiments were marked with an asterisk (*p*-value < 0.05).

### 2.10. Over-Representation Analysis of Pathways and Gene Ontologies (GOs) 

The test associated with the hypergeometric distribution which is implemented in R was employed to calculate the over-representation of KEGG (Kyoto Encyclopedia of Genes and Genomes) pathways [24], which had been downloaded from the KEGG website in July 2020. GOs were analysed for over-representation via the R package Gostats [25].

### 2.11. Metascape Analysis 

Gene enrichment analyses of differential GO/KEGG terms, biological processes, etc. comparing UMK1_con and UMK1_PAN were performed using the software metascape (http://metascape.org, [26]). Exclusive gene-sets of UMK1_con and UMK1_PAN based on Venn analysis were used as data sources. The metascape software applied hierarchical clustering to display calculated significant GO terms into a tree, which was spread into term clusters with a 0.3 kappa score as a threshold. The top enrichment clusters were represented as heatmaps with a colour scale ranging from grey to dark orange. Statistical significance was hereby displayed in dark orange and lack of enrichment in a grey colour. 

### 2.12. Kidney-Associated Cytokine Assay

Cell culture supernatants of untreated kidney organoids and kidney organoids treated with PAN (2 days after treatment) were kept for cytokine array. Relative expression levels of specific human kidney-associated and urinary proteins were determined using the Human Kidney Biomarker Array Kit from R&D Systems. The cytokine assay was implemented as recommended by the manufacturer. In brief, membranes were blocked for 1 h on a rocking platform. Prepared samples were incubated in the Detection Antibody Cocktail for 1 h at room temperature. Thereafter, the antibody–sample mixtures were pipetted onto the membranes and were incubated overnight at 2–8 °C on a rocking platform. The membranes were washed thoroughly and Streptavidin-HRP was added onto the membranes, which were incubated for 30 min at room temperature. ECL detection reagent was used to detect the spots on the membrane. 

### 2.13. Image and Data Analysis of the Kidney Cytokine Assay

Untreated organoids and organoids treated with PAN, which had been subjected to analysis with the kidney cytokine assay Human Kidney Biomarker Array Kit (R&D Systems) and scanned, were image-analysed with ImageJ [27]. The Microarray Profile plugin by Bob Dougherty and Wayne Rasband (https://www.optinav.info/MicroArray_Profile.htm) was employed to localize and quantify all spots on the array. As read-out, the integrated density generated by the Microarray profile plugin function Measure RT was used. In R/Bioconductor [28], the data resulting from the quantification was normalized with the Robust Spline Normalization from the Bioconductor lumi-package [29].

## 3. Results

### 3.1. Kidney Organoids Possess Structured Lobes with Distinct Tubular and Basement Membrane Structures

**Figure 1:**
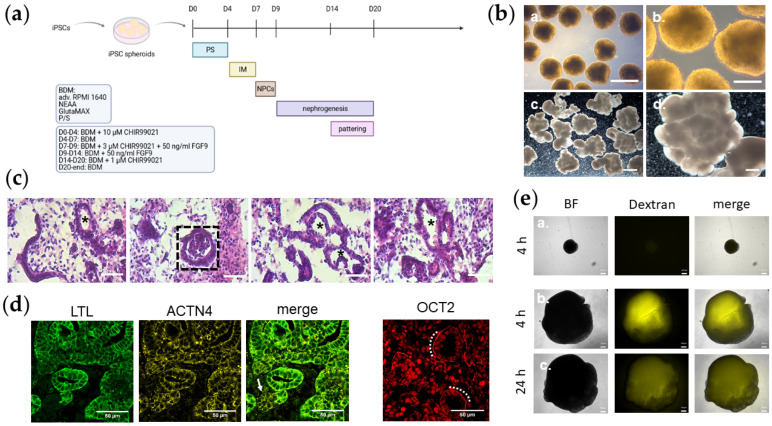
Lobular kidney organoids contain distinct kidney structures. (**a**) Schematic depiction of the protocol for generating kidney organoids. (**b**) Overview of iPSC spheroids at D8 after generation (a.,b.) and kidney organoids UMK2 at D21 (c.,d.) with binocular. (a.) 4× magnification under light microscope. Scale bar depicts 500 µm. (b.) 10× magnification under light microscope. Scale bar depicts 200 µm. (c.) 1× magnification. Scale bar depicts 2000 µm. (d.) 4× magnification. Scale bar depicts 2000 µm. (**c**) Morphology of organoid section via histological H&E staining. A typical glomerulus-like structure is depicted by a dashed rectangle. Tubule-like structures are marked with an asterisk. Scale bar depicts 50 µm. (**d**) Confocal pictures of glomerular (ACTN4, yellow) and tubular (LTL, green) structures in UMK1 sections. Nuclear OCT2 (*POU2F2*) (red) is expressed in UMK1 sections. A glomerulus-like structure was marked with an arrow. Scale bar depicts 50 µm. (**e**) Monitoring of iPSC spheroids and kidney organoids in a dextran uptake assay. (a.) iPSC spheroids treated with dextran after 4 h pulse. (b.) kidney organoids treated with dextran after 4 h pulse. (c.) kidney organoids treated with dextran after 24 h chase. Scale bar depicts 200 µm.

Kidney organoids of three iPS cell lines in duplicates were generated in approximately 20 days (Figure 1a; Table 1*)*. In contrast to the round-shaped iPSC spheroids, kidney organoids acquired a lobular morphology (Figure 1b). H&E staining of kidney organoid sections revealed structures morphologically similar to renal tubules and glomeruli (Figure 1c; Figure S1a). The tubule-like structures are marked with an asterisk and glomeruli framed with a dashed square (Figure 1c; Figure S1a). The presence of tubule-like structures was also detected by LTL and α-Actinin 4 (ACTN4) staining (Figure 1d). A structure at the rear end, which was negative for LTL but positive for ACTN4, was morphologically similar to a glomerulus and is marked with an arrow (Figure 1d). Additionally, the tubule-like structures were positive for an antibody against the Organic Cation Transporter 2 (OCT2), which marks the tubular plasma membranes (highlighted with a dotted line) (Figure 1d). The functional activity of the kidney organoids was shown with a dextran uptake assay (Figure 1e). A strong fluorescent signal was detected in kidney organoids incubated with dextran for a 4 h pulse (Figure 1e). In comparison, no signal was detected in dextran-treated iPSC spheroids (Figure 1e). After a 24 h chase, kidney organoids still showed a fluorescent signal (Figure 1e).

### 3.2. iPSC Spheroids Lose Pluripotent Gene Expression and Gain Kidney-Associated Genes during Differentiation

**Figure 2:**
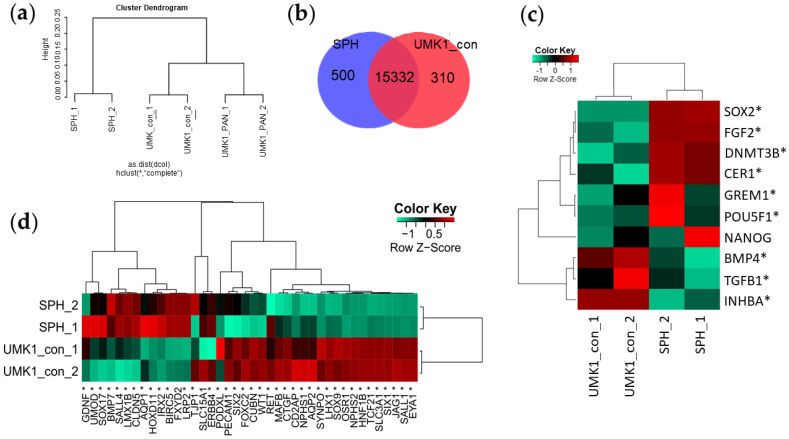
Comparative analysis of gene expression in iPSC spheroids and kidney organoids. (**a**) Similarities between spheroids and non-treated and PAN-treated organoids are shown in the cluster dendrogram. Control and PAN-treated UMK1 cluster together, and iPSC spheroids cluster separately. (**b**) The common gene-sets between spheroids and untreated kidney organoids consists of 15,332 genes. In total, 500 genes are exclusively expressed in spheroids and 310 genes in control organoids. (**c**) Expression of pluripotency-associated genes in UMK1_con and SPH depicted in a Pearson heatmap. (**d**) Expression of kidney-associated genes in UMK1_con and SPH depicted in a Pearson heatmap.

Global gene expression was investigated employing RNA Microarray Analysis. RNA of UMK1 kidney organoids, PAN-induced (PAN) and untreated control (con) were prepared in technical duplicates. Along with the aforementioned samples, RNA of iPSC spheroids (SPH) of the same genetic background as UMK1 was also analysed. A cluster dendrogram demonstrated similarities between UMK1_con and UMK1_PAN, whereas the duplicates of SPH clustered separately (Figure 2a). In order to control for successful differentiation into the kidney, we compared the gene-sets between SPH and UMK1_con. This revealed a total common gene-set of 15332 genes (Figure 2b). In total, 500 genes were exclusively expressed in SPH, and 310 exclusive genes were expressed in UMK1_con (Figure 2b). The common gene-set included upregulated and kidney-related GO terms such as “urogenital system development”, “mesonephric epithelium development”, “ureteric bud development”, “renal tubule development” and “nephron epithelium development” (see Supplementary File S1). A heatmap analysis displays the expression of pluripotency-associated genes in UMK1_con and SPH (Figure 2c). The genes *SOX2*, *FGF2, DNMT3B, CER1, GREM1* and *POU5F1* were expressed in SPH, whereas expression of the genes *BMP4, TGFB1 and INHBA* was observed in UMK1_con (Figure 2c).

The differentiation of iPSC spheroid towards kidney organoids was confirmed with a heatmap analysis composed of genes of early and late nephrogenesis (Figure 2d). Genes typical of early kidney development—*GDNF, SOX17, BMP7, SALL4, LMX1B* and *HOXD11*—were expressed in SPH, while podocyte-associated genes—*NPHS1*, *NPHS2*, *PECAM1*, *PODXL* and *SYNPO*—as well as renal-tubule-related genes—*AQP2* and *CUBN*—were expressed in UMK1_con (Figure 2d).

### 3.3. PAN Negatively Affects Podocytes and Partly Tubular Cells

**Figure 3:**
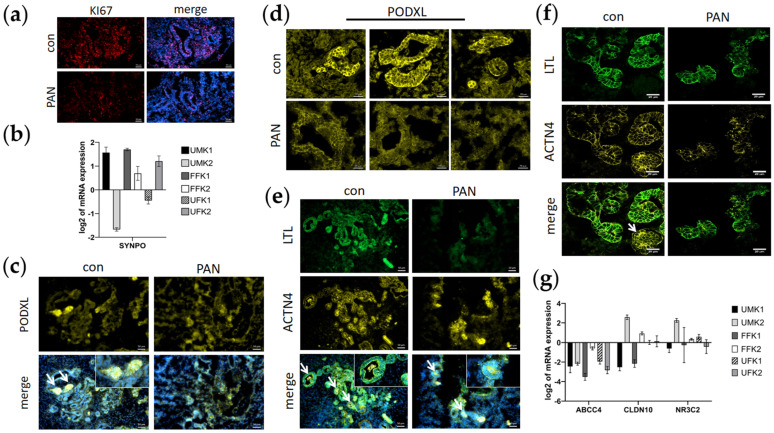
PAN leads to less defined glomerular and tubular structures. (**a**) PAN induction leads to a decrease in the number of highly proliferative KI67+ cells (red). Nuclei were stained with Hoechst33342 (blue). Scale bar depicts 50 µm. (**b**) Expression of the podocyte marker *SYNPO* is upregulated (UMK1, FFK1, FFK2, UFK2) after PAN treatment. Error bars depict standard error. (**c**) PODXL+ glomeruli (yellow) are less defined after PAN treatment. Glomeruli are marked with a white arrow. Nuclei were stained with Hoechst33342 (blue). Scale bar depicts 50 µm. (**d**) Confocal microscopy pictures of PODXL+ glomeruli with and without PAN. Scale bar depicts 20 µm. (**e**) LTL+ proximal tubules (green) and ACTN4+ glomeruli (yellow) are less defined after PAN treatment. Glomeruli are marked with a white arrow. Nuclei were stained with Hoechst33342 (blue). Scale bar depicts 50 µm. (**f**) Comparative confocal pictures of ACTN4 (yellow) and LTL-stained (green) organoid sections treated with and without PAN. Scale bar depicts 20 µm. Glomeruli are marked with a white arrow. (**g**) Expression of the tubular markers *ABCC4*, *CLDN10* and *NR3C2* are downregulated by PAN. Error bars depict standard error.

The kidney organoids sustained a high proliferation rate during the entire differentiation process. Immunofluorescence-based analysis of organoid sections detected proliferating KI67-positive cells, which were found to be reduced in organoid sections treated with PAN (Figure 3a).

Gene expression analysis revealed the upregulated expression of the podocyte-associated gene *SYNPO* in UMK1, FFK1, FFK2 and UFK2 kidney organoids (Figure 3). The podocyte marker PODXL was expressed by glomerular-like cells at the rear ends of the tubule-like structures (Figure 3; Figure S1b). Treatment with PAN resulted in a reduction in PODXL-expressing cells, and glomerular-like structures appeared to be less defined (Figure 3). The blurred boundaries of glomerular-like structures induced by PAN were closely observed by confocal microscopy (Figure 3). Additionally, tubular-like structures were found to be LTL-positive, and their rear ends were positive for ACTN4 (Figure 3). Reduced numbers of LTL^+^ and ACTN4^+^ cells were observed in PAN-treated organoid sections (Figure 3). In addition, the expression of the tubular markers—*ABCC4, CLDN10* and *NR3C2*—was downregulated in all organoid batches except for UMK2 (Figure 3). PAN treatment was found to negatively affect glomerular-like cells and partially affect tubular-like cells as well. 

### 3.4. Transcriptome Analysis Reveal Kidney-Related GO Terms Are Expressed in Urine-Stem-Cell-Derived iPSC Spheroids and Kidney Organoids

**Figure 4:**
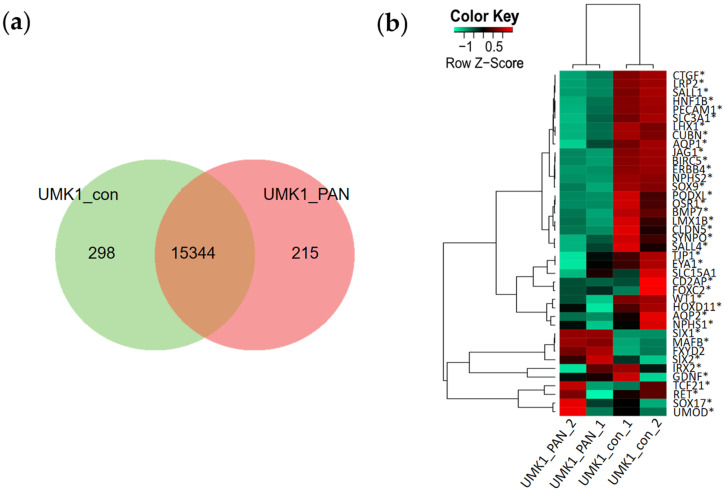
Transcriptome analysis of UMK1 with and without PAN treatment. (**a**) The common gene-sets between UMK1_con and UMK1_PAN consisted of 15344 genes. Exclusively expressed in UMK1_con and UMK1_PAN are 298 and 215 genes, respectively. (**b**) Expression of kidney-associated genes in UMK1_con and UMK1_PAN is displayed in a Pearson heatmap.

A Venn diagram comparison between UMK1_con and UMK1_PAN revealed a common gene-set of 15344, 298 exclusive genes in UMK1_con and an exclusive gene-set of 215 in UMK1_PAN (Figure 4). The top ten GO biological pathways of the exclusive UMK1_PAN gene-set are further described in Table S5 (see Supplementary File S2).

The expression of genes associated with nephrogenesis was compared between UMK1_con and UMK1_PAN with a Pearson heatmap (Figure 4b). Expression of the podocyte-associated genes *NPHS1, NPHS2*, *PECAM1*, *PODXL* and *SYNPO* as well as the tubular genes *SLC3A1, AQP1, CUBN* and *CLDN5* was observed in UMK1_con but was downregulated in UMK1_PAN (Figure 4b).

### 3.5. PAN Induces DNA Damage in Kidney Organoids

**Figure 5:**
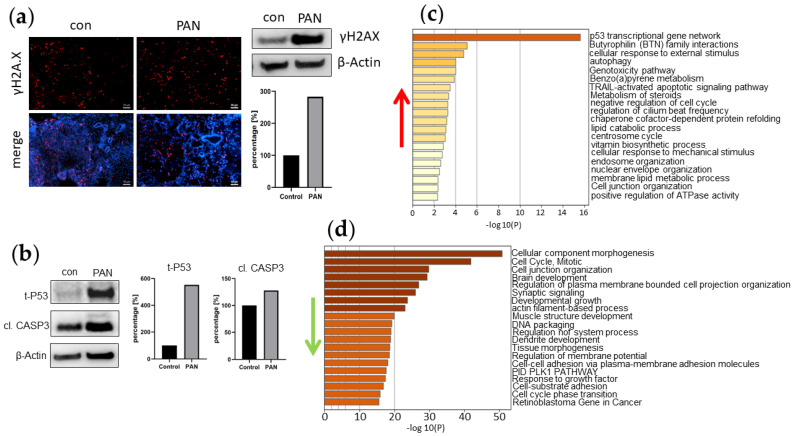
PAN treatment induces DNA damage in kidney organoids. (**a**) Elevated expression of γH2A.X (red) in PAN-treated kidney organoids UMK1. Nuclei were stained with Hoechst33342 (blue). Scale bar depicts 50 µm. (**b**) Elevated expression of t-P53 and cleaved Caspase 3 in PAN-treated kidney organoids UMK1. (**c**) Upregulated enrichment clusters include cell damage. (**d**) Cell-cycle-related enrichment clusters were downregulated by PAN treatment.

DNA damage in PAN-induced kidney organoids was evaluated by immunofluorescence and Western blot analysis. The DNA damage marker γH2A.X was expressed in both untreated and PAN-treated kidney organoid sections (Figure 5a). Interestingly, quantitative Western blot analysis revealed a three-fold increase in γH2A.X protein expression levels in PAN-induced UMK1 compared to an untreated control (Figure 5a). DNA damage induced by PAN was also observed by the detection of total P53 protein, which was expressed at higher levels in PAN-treated UMK1 than in the controls (Figure 5b). Additionally, a higher amount of cleaved Caspase 3 protein was observed upon PAN treatment (Figure 5b). We could also observe higher amounts of γH2A.X, P53 and cleaved Caspase 3 protein in the other PAN-treated kidney organoid batches—UMK2, FFK1, FFK2, UFK1 and UFK2 (Figure S1c). However, in the UFK2 organoids, we observed reduced levels of γH2A.X and cleaved CASP3 after PAN treatment (Figure S1c). Further indicators of cell damage were also observed in upregulated GO enrichment clusters such as “p53 transcriptional gene network”, “autophagy”, “TRAIL-activated apoptotic signalling pathway” and “negative regulation of cell cycle” (Figure 5c). 

Additionally, other downregulated cell-cycle-related GO enrichment clusters included “mitotic cell cycle” and “developmental growth” (Figure 5d). Besides other effects caused by PAN, the substance is known to damage podocytes, which particularly affects the podocyte membranes. With regard to this, we observed the downregulation of the GO terms “cell junction organization”, “regulation of plasma membrane bounded cell projection organization”, “actin-filament-based process” and “cell–cell adhesion via plasma-membrane adhesion molecules” (Figure 5d). Similarly, a KEGG pathway analysis revealed the downregulation of genes associated with “cell cycle” (Table S6; Supplementary File S3), as well as upregulated genes within the “P53 signalling pathway” (Table S7; Supplementary File S4). Treating kidney organoids UMK1 with PAN was associated with the regulation of various biological pathways. We especially concentrated on the 215 genes solely expressed by the PAN-treated UMK1 organoids as seen in the Venn analysis (Figure 4). With a detection p-value below the threshold of 0.05, we focused on the top 10 expressed GO biological pathways, which included: “lipoxygenase pathway”, “inflammatory response”, “establishment of skin barrier”, “hepoxilin biosynthetic process”, “regulation of adaptive immune response based on somatic recombination of immune receptors built from immunoglobulin superfamily domains”, “lactate transmembrane transport”, “response to biotic stimulus”, “establishment of localization”, “multicellular organismal water homeostasis” and “regulation of cytokine production” (Table S5). Interestingly, pathways involved in inflammation and immune responses were significantly regulated in kidney organoids treated with PAN. We could also confirm the transcriptome data by qRT-PCR, which demonstrated an upregulation of genes associated with the lipoxygenase pathway (*ALOX12B*) and P53 signalling pathway (*SESN2*, *FAS*) and a downregulation of cell-cycle-related genes (*CCNB2*, *PLK1* and *BUB1*) (Figure S2a).

### 3.6. Inflammation-Associated Processes Are Elevated in PAN-Induced Kidney Organoids

**Figure 6:**
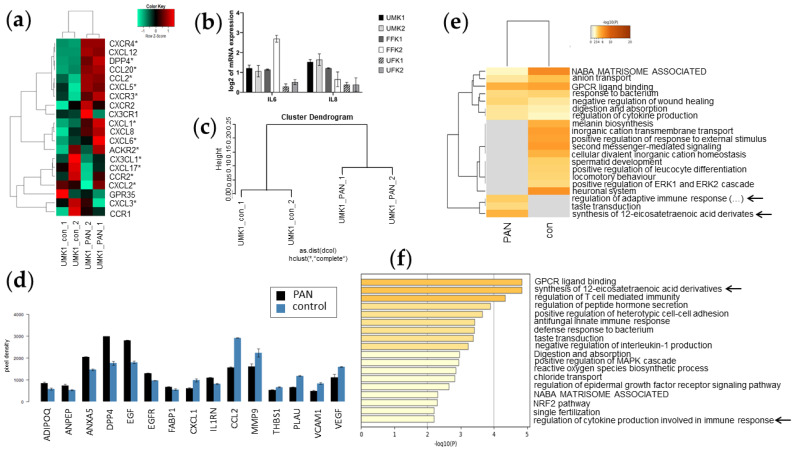
Inflammation-associated gene expression and cytokine secretion in untreated and PAN-treated kidney organoids. (**a**) Pearson heatmap of the expression of immune-related genes in control and PAN treatment. (**b**) Expression of IL-6 and IL-8 is elevated in PAN-induced kidney organoids as measured by qRT-PCR. Error bars depict standard error. (**c**) Cluster dendrogram with the technical duplicates of untreated kidney organoids clustering together and the PAN-treated kidney organoids cluster separately. (**d**) Cytokine array data comparing expression between untreated control and PAN treatment. (**e**) Metascape-generated heatmap comparing UMK1_PAN and UMK1_con included inflammation- and immune-response-related GOs. Subjected gene-sets are based on the Venn analysis in Figure 4. (**f**) Bar graph of non-redundant enrichment clusters after PAN treatment.

As the transcriptome analysis revealed indications that PAN induces inflammation and acute immune responses, we analysed the expression of markers of the myeloid cell lineage via Pearson heatmap analysis (Figure 6a). Various pro-inflammatory –CXC- and –CC- chemokines as well as DPP4—an established marker of the proximal tubular compartment—were expressed in UMK1_PAN but not in UMK1_con (*CXCR4*, *DPP4*, *CCL20*, *CCL2*, *CXCL5*, *CXCR3*, *CXCL1* and *CXCL6*) (Figure 6a). In contrast, anti-inflammatory –CXC- and –CC- chemokines were primarily expressed in UMK1_con and not UMK1_PAN (*CX3CL1*, *CXCL17*, *CCR2*, CXCL2 and *CXCL3*) (Figure 6a). 

The expression of pro-inflammatory cytokines—*IL8* and *IL6*—was upregulated in all organoid batches, except for UMK1, after PAN treatment compared to their specific controls (Figure 6b). Cytokine secretion of PAN-induced UMK1 was additionally analysed using a “Human Kidney Biomarker array” and compared to an untreated control. A cluster dendrogram based on the cytokine array data showed clustering of the duplicates control versus PAN treatment (Figure 6c). Upon PAN treatment, the concentrations of ADIPOQ, ANPEP, ANXA5, DPP4, EGF, EGFR and IL1RN increased significantly *(*Figure 6d). In contrast, CXCL1, CCL2, MMP9, THBS1, PLAU and VCAM1 were secreted at lower levels compared to untreated controls (Figure 6d). 

The exclusive gene-sets of UMK1_con (298 genes) and UMK1_PAN (215 genes) from the Venn analysis in Figure 4 were subjected to metascape-based analysis (Figure 5e,f and Figure 6). When comparing the enriched gene clusters of UMK1_con and UMK1_PAN, the gene clusters “regulation of adaptive immune response based on somatic recombination of immune receptors built from immunoglobulin superfamily domains” and “synthesis of 12-eicosatetraenoic acid derivates” emerged in UMK1_PAN (Figure 6e). These fatty acids are involved in inflammatory processes and thus implying that PAN induces inflammatory responses in the kidney organoids. The analysis of non-redundant enrichment clusters additionally supported the observation that PAN treatment induces inflammation and immune response as the enrichment clusters comprised “synthesis of 12-eicosatetraenoic acid derivates“ and “regulation of cytokine production involved in immune response” (Figure 6f). 

### 3.7. PAN Activates the Renin–Aldosterone–Angiotensin System (RAAS)

**Figure 7:**
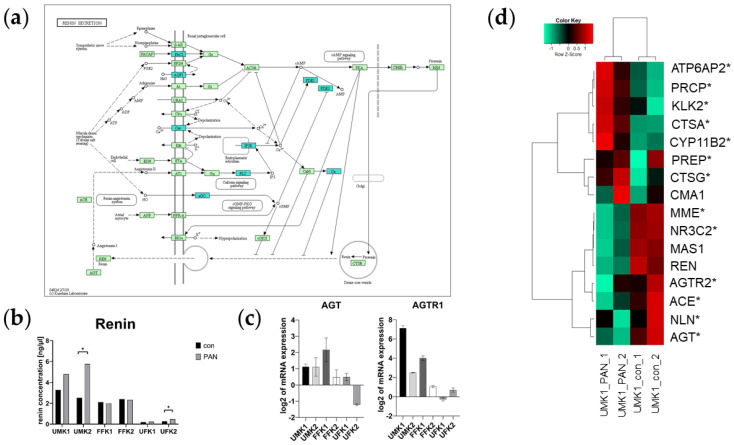
PAN induction affects the RAAS in kidney organoids. (**a**) PAN induces downregulation of genes of the KEGG pathway renin secretion. Downregulated genes are marked in blue. (**b**) Renin concentration (ng/µL) in conditioned media of untreated and PAN-treated kidney organoids. Significance is determined by α-value ≤ 0.05. (**c**) Expression of RAAS-associated AGT and AGTR1 is upregulated in PAN-induced kidney organoids. Error bars depict the standard error. (**d**) Pearson’s heatmap depicting the expression of RAAS-associated genes in untreated and PAN-treated kidney organoids.

Kidney injury, especially glomerular damage, is associated with altered blood pressure. We examined this by investigating the renin–aldosterone–angiotensin System (RAAS). The transcriptome data revealed PAN-induced downregulation of the renin-secretion-associated genes—*PAC1, AQP1, Cav, IP3R, PLC, sGC, PDE1, PDE3* and *Cn* (Figure 7a). The secretion of renin by the kidney organoids was measured using a commercially available renin assay kit. An increase in renin secretion after PAN treatment was observed in the organoid batches UMK1, UMK2 and UFK2, whereas statistical significance with respect to the control was only found in UMK2 and UFK2 (α-value ≤ 0.05) (Figure 7b). No significant difference was observed between control and PAN treatment in FFK1, FFK2 and UFK1 organoids (Figure 7b). The mRNA expression of the key genes of the RAAS pathways—*AGT* and *AGTR1*—was upregulated in almost all kidney organoids (Figure 7c). AGT was downregulated in PAN-treated UFK2 organoids and *AGTR1* was downregulated in PAN-treated UFK1 organoids (Figure 7c). In the Pearson’s heatmap of genes involved in RAAS, we could survey a section in which the genes *MME*, *NR3C2*, *AGTR2*, *ACE* and *AGT* were mostly expressed in UMK1_con (Figure 7d). On the other hand, the genes *ATP6AP2*, *PRCP*, *KLK2 and PREP* were mainly expressed in UMK1_PAN (Figure 7d).

## 4. Discussion

In this study, we demonstrated the successful generation of kidney organoids. We used three iPS cell lines of distinct genetic background in technical duplicates, of which two were derived from urine-derived renal progenitor cells [16,17] and one from foetal foreskin fibroblast cells [18] (see Table 1). Kidney damage was modelled by stimulating kidney organoids for 48 h with 50 µg/mL of the nephrotoxic substance puromycin aminonucleoside (PAN). 

In our previous studies, we observed that urine-derived renal progenitor cells (UdRPCs) are MSCs and additionally could be differentiated into tubular- and podocyte-like cells [17,30]. Interestingly, we observed that UdRPC-derived iPSCs spheroids (SPH) and untreated kidney organoids (UMK1_con) shared a common set of 15332 expressed genes, while they clustered distinctly. From this common gene-set, we observed upregulated genes associated with kidney-related GO terms such as “urogenital system development”, “mesonephric epithelium development”, “ureteric bud development”, “renal tubule development” and “nephron epithelium development”. Additionally, our heatmap analysis revealed that a set of genes associated with kidney development was expressed in both untreated kidney organoids as well as iPSC spheroids (e.g., *CUBN*, *NPHS1, NPHS2* and *SYNPO*). Additional and deeper investigations regarding the potential of UdRPCs is beyond the scope of the current study and should be conducted in the future.

While comparing kidney organoids with iPSC spheroids, we observed that kidney organoids had a more lobular morphology in comparison to the typical round morphology of iPSC spheroids. Moreover, H&E stainings as well as immunofluorescence-based stainings showed the emergence of tubular- as well as glomerular-like structures within our kidney organoids. Previous studies demonstrated the ability of proximal tubules to take up dextrans within hPSC-derived kidney organoids [19,31]. Therefore, the functionality of our kidney organoids was evaluated with a dextran uptake assay. Endocytosis of dextran was demonstrated in our kidney organoids, whereas iPSC spheroids did not take up dextran. As previously mentioned, the kidney organoids are composed of LTL- and OCT2-positive tubular- and glomeruli-like structures, which express PODXL and ACTN4. PODXL is expressed on the podocyte cell surface [32] and is involved in the formation of the slit diaphragm, while ACTN4 co- localizes with cytoskeleton filaments within the foot processes [33]. Stimulation with the nephrotoxic purine antagonist PAN interestingly led to the upregulation of *SYNPO* expression. However, our transcriptome data suggest PAN-induced podocyte damage, as we found the increased expression of podocyte-associated factors such as *NPHS2* and *PODXL* in UMK1_con and reduced expression in UMK1_PAN. Corroborating our findings, Luimula et al. [34] and Lee et al. [35] observed reduced NEPHRIN expression in regions of foot processes effacement after PAN injection in rats. *NPHS1* mutation or reduced NEPHRIN expression was found to be involved in various glomerular diseases [35,36]. A lack of filtration of proteins due to a defect in the slit diaphragm results in proteinuria [37], which can eventually lead to reversible podocyte detachment from the glomerular basement membrane (GBM) [7]. Downregulated enrichment clusters “cell junction organization”, “regulation of plasma membrane bounded cell projection organization”, “actin-filament-based process” and “cell–cell adhesion via plasma-membrane adhesion molecules” imply that PAN treatment interferes with cell–cell contact. Based on these findings, we concluded that our PAN-treated kidney organoids can be used to model glomerular-associated diseases. 

The kidney organoids contained a high number of KI67^+^ -proliferative cells, which were reduced by PAN induction. KI67 expression is found in cells during the interphase of the cell cycle but is absent in the G0 phase [37]. Adult kidney cells have a slow proliferation rate [38]. Especially glomerular cells, such as podocytes, are in the postmitotic G0 phase [38]. Therefore, we suspect that our kidney organoids are rather immature and similar to findings of previous studies, where hPSC-derived kidney organoids resemble human foetal kidneys [31,39,40]. Besides the reduction in KI67-positive cells, we observed upregulation in the enrichment terms “negative regulation of cell cycle” and downregulation of “mitotic cell cycle” and “developmental growth “in PAN-treated kidney organoids. In addition, the levels of t-P53 protein increased in all PAN-treated organoid batches, and an enrichment of the KEGG pathway “p53 transcriptional gene network” was observed. Therefore, we assumed a progression from interphase to G0 resulting in cell cycle arrest in our PAN-treated kidney organoids. 

DNA damage triggered by PAN treatment might be the reason for the observed cell cycle arrest. Even though we did not observe a difference in the level of γH2AX between the control and PAN-treated UMK1 in the IF-based staining, we found a 2-fold higher amount of protein in PAN-treated UMK1 than in the untreated control. A higher level of γH2AX was also observed in the other organoid batches—UMK2, FFK1, FFK2 and UFK1. PAN treatment induced increased apoptosis in the kidney spheroids—UMK1, UMK2, FFK1, FFK2 and UFK1—which was detected by increased levels of cleaved Caspase 3 in PAN-treated kidney organoids. This finding was also observed by Kang et al. [8], where they detected active Caspase 3 in PAN-induced podocytes [8]. In addition, they found autophagy was induced by PAN prior to apoptosis [8]. Similar to their observation, in our kidney organoid model, enriched clusters of “autophagy” and “TRAIL-activated apoptotic signalling pathway” were upregulated upon PAN treatment. Interestingly, less γH2AX and cleaved Caspase 3 was found in PAN-treated UFK3 organoids. This finding can be attributed to the variability in batches of organoids. There are manifold reasons for a high variability between organoids, depending on the employed cell lines or the heterogeneity of clones and the genotypes [12]. As the other organoid batches showed the same tendencies regarding increased levels of γH2AX and cleaved Caspase 3, we conclude that PAN treatment results in DNA damage and the activation of cell-death-associated processes. 

Besides the induction of DNA damage, we also observed the upregulation of inflammatory processes upon PAN treatment. The pro-inflammatory regulators *IL-6* and *IL-8* as well as the expression of other pro-inflammatory genes such as *CXCR4*, *CXCL12*, *DPP4*, *CCL20*, *CCL2*, *CXCL5*, *CXCR3*, *CXCR2*, *CX3CR1*, *CXCL1*, *CXCL8* and *CXCL6* were upregulated in PAN-treated UMK1. From the results of the cytokine array, we observed the secretion of the reno-protective cytokines ADIPOQ, Diannexin (ANXA5), EGF and EGFR. These cytokines are known to protect against renal ischemia and reperfusion injury as well as to promote recovery after AKI [41,42]. Moreover, ADIPOQ levels are used as a prognostic tool for ESRD [42]. We therefore propose that PAN treatment causes renal inflammation and as a consequence, the activation of protective and anti-inflammatory reactions within cells. On the other hand, previous studies have demonstrated that tubular epithelial cells express the pro-inflammatory Interleukin-1α, which is involved in immune response [43]. This cytokine induces local inflammation via chemokine secretion, subsequently attracting neutrophils, macrophages and lymphocytes [43]. Signalling is dependent on the IL-1 receptor, which can be blocked by the anti-inflammatory antagonist IL1RN [43,44]. This led us to the assumption that the higher amounts of IL1RN in the secretome of PAN-treated kidney organoids functioned as a counteraction to the induced inflammation. Likewise, our transcriptome data suggest a cytokine-induced immune reaction in PAN-treated kidney organoids as the non-redundant enrichment cluster “regulation of cytokine production involved in immune response” was unveiled. 

Moreover, the untreated kidney organoids primarily expressed anti-inflammatory genes such as *CX3CL1*, *CXCL17*, *CCR2*, *CXCL2*, *GPR35*, *CXCL3* and *CCR1*, which might imply a form of protection against inflammatory processes in the control kidney organoids UMK1_con. 

An inflammation response upon PAN treatment was additionally detected in the metascape-based analysis with the enrichment clusters “synthesis of 12-eicosatetraenoic acid derivates” and “regulation of adaptive immune response based on somatic recombination of immune receptors built from immunoglobulin superfamily domains”. Moreover, we found “synthesis of 12-eicosatetraenoic acid derivates” to be the top non-redundant enrichment cluster in UMK1_PAN. Eicosatetraenoic acids are metabolites from the lipoxygenase pathway, which we observed as one of the top GO biological pathways within the 215 exclusive genes of the PAN-treated UMK1 organoids. Eicosanoids are synthesized from polyunsaturated fatty acids such as arachidonic acids [45,46]. Lipoxygenases synthesize 5-, 12-, or 15-hydroxyeicosatetraenoic (HETE) acids from arachidonic acids [47]. They are involved in various metabolic processes such as cellular inflammation [45,46]. It was recently discovered that lipoxygenases play a role in kidney damage of diabetic nephropathy and their synthesised products induce the synthesis of stimulants involved in kidney fibrosis [47]. One of the lipoxygenases is the 5-lipoxygenase (*ALOX5*), which is involved in inflammatory diseases [48]. Previous reports suggest that inhibiting 5-lipoxygenase led to a reduction in renal fibrosis and CKD progression [48]. Additionally, the synthesis product 5-HETE stimulates T-cell production, one of the major immune cells [47]. In addition, it has been described that 12-lipoxygenase and 15-lipoxygenase synthesize 12-HETE and 15-HETE, which contribute to the overexpression of pro-inflammatory cytokines in macrophages [47]. We detected the elevated expression of 12-lipoxygenase in PAN-treated kidney organoids and together with the findings of our transcriptome data, it suggests that PAN stimulation leads to an increase in inflammation and other injury-associated processes. We conclude that PAN induction of pro-inflammatory processes in kidney organoids can be considered for modelling inflammation-associated acute kidney injury and associated diseases. 

Due to their inherent transport activity, proximal tubules are susceptible to damage by kidney injury, renal ischemia and nephrotoxicity [49]. LTL staining detected proximal tubules in our kidney organoids, and we observed these to be partially affected by PAN induction. Structurally, tubules of PAN-induced kidney organoids had partly disrupted cell membranes, and tubular-associated genes such as *ABCC4*, *CLDN10* and *NR3C2* were downregulated. Additionally, our transcriptome data revealed the low-level expression of tubule-associated genes such as *CUBN* and *AQP1* in PAN-treated UMK1 organoids compared to the control. Similar observations of tubular damage and stimulated glomerular nephropathy were made in rats when PAN was administered chronically [50]. Additionally, PAN-treated kidney organoids secreted significantly higher amounts of FABP, ANPEP (CD13) and DPP4 (CD26), which are associated with the proximal tubule compartment. 

Within the proximal tubular compartment, L-FABP is capable of binding to lipid peroxide and protects from oxidative stress [51]. However, tubular damage results in the secretion of L-FABP into urine [51]. The proteins ANPEP and DPP4 are expressed in the apical brush border epithelium of proximal tubules and are shed into urine upon increased tubular stress, caused by various renal injuries [52]. This may lead to an impairment of the glomerular filtration rate and thus a shedding of glomerular proteins [52]. The higher amounts of proteins in urine eventually exceed the capacity of proximal tubules to reabsorb proteins, which further adds up to proteinuria [52]. Since the aforementioned proteins are cumulatively secreted into urine upon renal injuries, this was an indication that our kidney organoids can be used for kidney injury models, specifically the proximal tubular compartment. 

We observed that PAN had an effect on the renin–angiotensin–aldosterone system (RAAS). The RAAS has a key role in the maintenance of blood pressure and body fluid homoeostasis. In the kidney, the RAAS is essential for normal development, and complications can lead to congenital anomalies of the kidney and the urinary tract (CAKUT) [53]. A heatmap analysis revealed the expression of *ATP6AP2*, *PCRP*, *KLK2* and *PREP* in our PAN-treated, but not in the untreated kidney organoids. Upon pathological conditions such as renal dysfunction, previous studies observed the activation of pro-inflammatory and pro-fibrotic molecules by ATP6AP2 [54]. Similarly, KLK2 plays a major role in inflammatory kidney diseases, where it is involved in various physiological processes [55]. On the other hand, PCRP and PREP have reno-protective properties [56,57]. While PRCP degrades ANGII to ANG (1–7) [56], studies in hypertensive rats revealed that PREP synthesizes anti-fibrotic molecules, which also decrease the infiltration of inflammatory cells [57]. 

In the untreated kidney organoids, we observed the expression of key components of the RAAS such as *AGT*, *ACE* and *AGTR2*, as well as *MMP* and *NR3C2*. MMP acts together with ACE2 for the production of ANG (1-7) to counteract against overproduced ANGII, which finally prevents hypertension and organ damage [58,59]. Even though NR3C2 is involved in the regulation of fluid, electrolytes and blood pressure homoeostasis [60], an over-activation can cause organ injury induced by inflammatory and fibrotic processes [61,62]. 

Interestingly, KEGG pathway analysis revealed the downregulation of renin-secretion-associated genes, mainly involved in the calcium and cGMP-PKG signalling pathways. Renin secretion is directly linked to cAMP formation, which is regulated by intracellular Ca^2+^ concentration [63]. High levels of Ca^2+^ correlate with decreased renin secretion [63]. Additionally, high levels of cGMP were also found to negatively affect renin metabolism [64]. The results of the renin secretion assay as well as the KEGG analysis revealed decreased calcium signalling and increased renin secretion in the PAN-treated kidney organoids. Previous studies suggest that increased renin and angiotensinogen (AGT) levels lead to an increased ANGII concentration, thereby stimulating hypertension and organ damage [65]. We made a similar observation, as we measured an upregulation of AGT expression in PAN-induced kidney organoids. Additionally, we demonstrated the upregulated expression of the ANGII receptor *AGTR1*. Besides its role in the regulation of blood pressure, AGTR1 is associated with various pathological conditions such as hypertension and diabetic nephropathy [66]. In conclusion, PAN treatment induced upregulated renin secretion, which subsequently increased ANGII and AGTR1 expression and which may lead to the emergence of pathological conditions such as high blood pressure under certain circumstances.

## 5. Conclusions

This study has revealed that human kidney organoids treated with puromycin aminonucleoside hold promise in the study of various pathological kidney conditions, which affect the glomerular and tubular regions. PAN induces immune response such as inflammation, DNA damage, apoptosis and cell death. Furthermore, PAN activated the RAAS pathway, therefore demonstrating the relevance in pro-inflammatory and reno-protective processes as well as the emergence of pathological kidney conditions such as ANGII-induced hypertension over time. In the future prospect, a deeper understanding of kidney-related disease mechanisms by harnessing kidney organoid models can be beneficial for novel drug discovery and development.

## Figures and Tables

**Table 1:** Representation of naming for all kidney organoid batches.

Description	Gender and Ethnicity	Age	Abbreviation
iPSC spheroids derived from urine cell UM51 iPSCs	Male, African	51	iPSC spheroids (SPH)
Kidney organoids derived from urine cell UM51 iPSCs, biological replicate 1	Male, African	51	UM51 kidney organoids_1 (UMK1)
Kidney organoids derived from urine cell UM51 iPSCs, biological replicate 2	Male, African	51	UM51 kidney organoids_2 (UMK2)
Kidney organoids derived from foetal foreskin (FF) iPSCs, biological replicate 1	Male, Caucasian	foetal	FF kidney organoids_1 (FFK1)
Kidney organoids derived from foetal foreskin (FF) iPSCs, biological replicate 2	Male, Caucasian	foetal	FF kidney organoids_2 (FFK2)
Kidney organoids derived from urine cell UF21 iPSCs, biological replicate 1	Female, Caucasian	21	UF21 kidney organoids_1 (UFK1)
Kidney organoids derived from urine cell UF21 iPSCs, biological replicate 2	Female, Caucasian	21	UF21 kidney organoids_2 (UFK2)

## Data Availability

All microarray data will be available at NCBI GEO under the accession number GSE186823 when the manuscript is accepted.

## Supplementary Materials

The following are available online at https://www.mdpi.com/2073-4409/11/4/635/s1, Table S1: Composition of basal spheroid medium, Table S2: Composition of basal differentiation medium, Table S3: List of utilized antibodies, Table S4: List of used qRT-PCR primers, Table S5: Top ten GO biological pathways expressed in PAN-treated kidney organoids, Table S6: Top ten downregulated biological pathways and processes of KEGG analysis, Table S7: Top ten upregulated biological pathways and processes of KEGG analysis, Figure S1: Effects of PAN on kidney organoids detected by H&E staining, immunofluorescence staining and Western blotting, Figure S2:Confirmation of transcriptome data and details of the cytokine array data. Supplementary File S1: Complete list of GO terms based on the common gene-set between SPH and UMK1_con_. Supplementary File S2: Complete list of regulated GO biological pathways in UMK1_PAN exclusive gene-set. Supplementary File S3: Complete list of downregulated KEGG pathways in the common gene-set of UMK1_con and UMK1_PAN. Supplementary File S4: Complete list of upregulated KEGG pathways in the common gene-set of UMK1_con and UMK1_PAN.
