# Correction: Nguyen et al. The Nephrotoxin Puromycin Aminonucleoside Induces Injury in Kidney Organoids Differentiated from Induced Pluripotent Stem Cells. *Cells* 2022, *11*, 635

**DOI:** 10.3390/cells11131980

**Published:** 2022-06-21

**Authors:** Lisa Nguyen, Wasco Wruck, Lars Erichsen, Nina Graffmann, James Adjaye

**Affiliations:** Institute of Stem Cell Research and Regenerative Medicine, Medical Faculty, Heinrich-Heine University, 40225 Dusseldorf, Germany; lisa.nguyen@med.uni-duesseldorf.de (L.N.); wasco.wruck@med.uni-duesseldorf.de (W.W.); lars.erichsen@med.uni-duesseldorf.de (L.E.); nina.graffmann@med.uni-duesseldorf.de (N.G.)

In the original publication [1], there was a mistake in the legend for Figure 1d. The staining of OCT2 in Figure 1d shows a staining of an antibody against the protein of the gene *POU2F2* and not the protein of *SLC22A2*, as intended. The correct legend appears below.

The authors apologize for any inconvenience caused and state that the scientific conclusions are unaffected. This correction was approved by the Academic Editor. The original publication has also been updated.

## Figures and Tables

**Figure 1:**
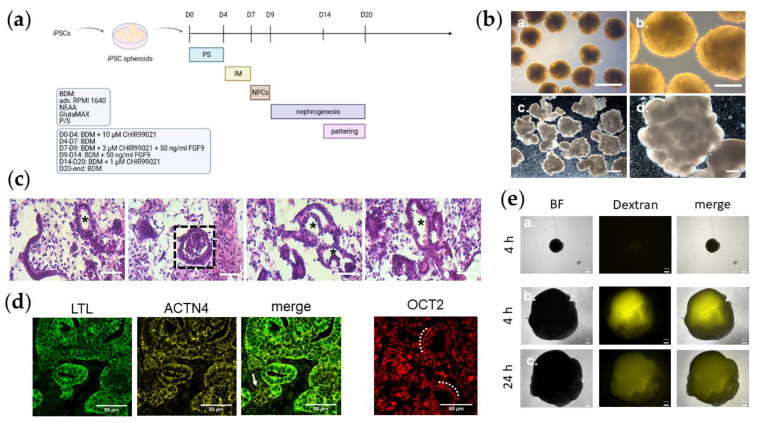
Lobular kidney organoids contain distinct kidney structures. (**a**) Schematic depiction of the protocol for generating kidney organoids. (**b**) Overview of iPSC spheroids at D8 after generation (a.,b.) and kidney organoids UMK2 at D21 (c.,d.) with binocular. (a.) 4× magnification under light microscope. Scale bar depicts 500 µm. (b.) 10× magnification under light microscope. Scale bar depicts 200 µm. (c.) 1× magnification. Scale bar depicts 2000 µm. (d.) 4× magnification. Scale bar depicts 2000 µm. (**c**) Morphology of organoid section via histological H&E staining. A typical glomerulus-like structure is depicted by a dashed rectangle. Tubule-like structures are marked with an asterisk. Scale bar depicts 50 µm. (**d**) Confocal pictures of glomerular (ACTN4, yellow) and tubular (LTL, green) structures in UMK1 sections. Nuclear OCT2 (*POU2F2*) (red) is expressed in UMK1 sections. A glomerulus-like structure was marked with an arrow. Scale bar depicts 50 µm. (**e**) Monitoring of iPSC spheroids and kidney organoids in a dextran uptake assay. (a.) iPSC spheroids treated with dextran after 4 h pulse. (b.) kidney organoids treated with dextran after 4 h pulse. (c.) kidney organoids treated with dextran after 24 h chase. Scale bar depicts 200 µm.
