# Supplementary figures and images for: The Nephrotoxin Puromycin Aminonucleoside Induces Injury in Kidney Organoids Differentiated from Induced Pluripotent Stem Cells
Source: Cells. 2022 Feb 11;11(4):635. doi: 10.3390/cells11040635 (PMC8870209; doi:10.3390/cells11040635)

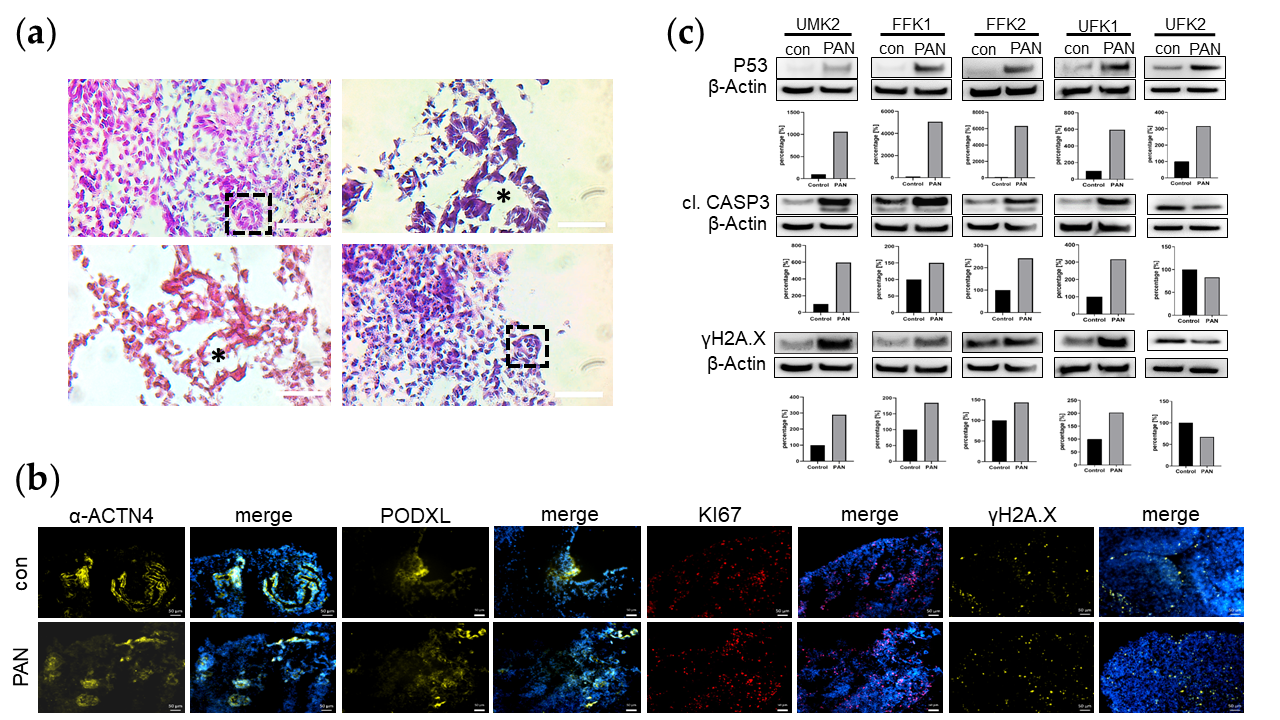

Supplement: Supplementary file 1 [file cells-11-00635-s001.zip › Figure S1.tif]

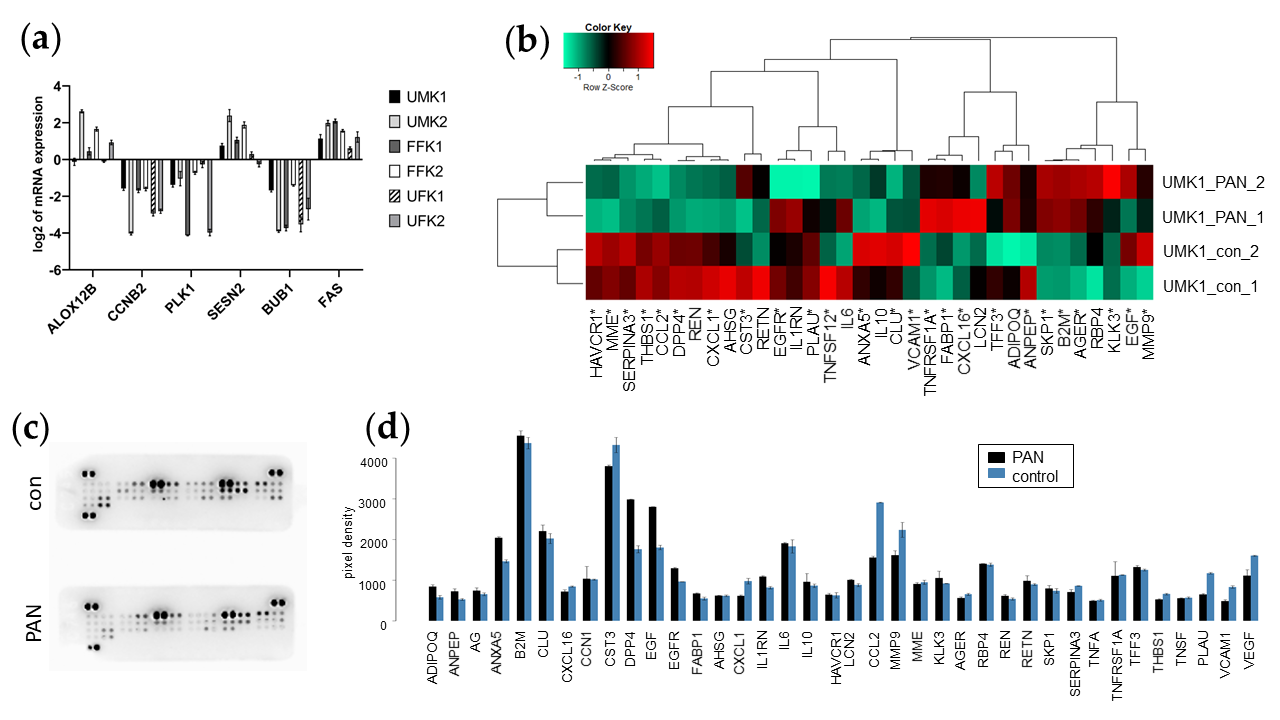

Supplement: Supplementary file 1 [file cells-11-00635-s001.zip › Figure S2.tif]
